# Impact of Immunogenetic IL28B Polymorphism on Natural Outcome of HCV Infection

**DOI:** 10.1155/2014/710642

**Published:** 2014-02-26

**Authors:** Valli De Re, Laura Gragnani, Elisa Fognani, Alessia Piluso, Francesco Izzo, Alessandra Mangia, Marina Crovatto, Graziella Gava, Pietro Casarin, Domenico Sansonno, Vito Racanelli, Salvatore De Vita, Pietro Pioltelli, Laura Caggiari, Mariangela De Zorzi, Massimiliano Berretta, Andrea Gini, Antonella Zucchetto, Franco Maria Buonaguro, Paolo De Paoli, Anna Linda Zignego

**Affiliations:** ^1^Bio-Proteomics Facility, Department of Translational Research, CRO, National Cancer Institute, 33081 Aviano, Italy; ^2^Interdepartmental Center for Systemic Manifestations of Hepatitis Virus MASVE, Department of Experimental and Clinical Medicine, University of Florence, 50121 Florence, Italy; ^3^Hepatobiliary Unit, National Cancer Institute “Fondazione Pascale”, 80138 Naples, Italy; ^4^Liver Unit, IRCCS Casa Sollievo della Sofferenza Hospital, 71013 San Giovanni Rotondo, Italy; ^5^Cytogenetics and Molecular Biology Unit, Santa Maria degli Angeli Hospital Pordenone, 33170 Pordenone, Italy; ^6^Internal Medicine-Liver Unit, Santa Maria degli Angeli Hospital Pordenone, 33170 Pordenone, Italy; ^7^Immunology Section, Department of Biomedical Sciences and Human Oncology, University of Bari Medical School, 70121 Bari, Italy; ^8^Clinic of Rheumatology, Department of Medical and Biological Sciences, University Hospital Santa Maria della Misericordia, 33100 Udine, Italy; ^9^Hematology and Transplant Unit, San Gerardo Hospital, University of Milano-Bicocca, Monza, Italy; ^10^Medical Oncology, Centro di Riferimento Oncologico, 33081 Aviano, Pordenone, Italy; ^11^Epidemiology and Biostatistics, Centro di Riferimento Oncologico, IRCCS, 33081 Aviano, Italy; ^12^Molecular Biology and Viral Oncology, National Cancer Institute “Fondazione Pascale”, 80138 Naples, Italy

## Abstract

With the aim of investigating whether interleukin 28B gene (IL28B) rs1297860 polymorphism is associated with different hepatitis C (HCV) infection statuses, we compared IL28B allelic distribution in an Italian case series of 1050 patients with chronic infection and different outcomes, 47 individuals who spontaneously cleared HCV, and 178 blood donors. Furthermore, we compared IL28B variants among 3882 Caucasian patients with chronic infection, 397 with spontaneous clearance, and 1366 blood donors reported in PubMed. Overall data confirmed a relation between IL28B C allele and HCV spontaneous clearance. Furthermore, we found that IL28B T allele had a weak relation with chronic HCV progression to hepatocellular carcinoma. Study findings are in accordance with the hepatocellular carcinogenic model where IL28B TT genotype, by promoting a persistent chronic hepatitis which leads to both hepatocyte injury and chronic inflammation, could facilitate HCC development. Conversely, patients with lymphoproliferative disorders had not any significantly different IL28B rs1297860 allelic distribution than those with chronic HCV, but, like all chronic HCV-related diseases, they showed a lower CC frequency than patients who spontaneously cleared HCV. Study results confirmed the model of persistent HCV infection as a risk factor for the pathogenesis of both liver and lymphoproliferative disorders.

## 1. Introduction

Hepatitis C virus (HCV), a positive-strand RNA virus widely diffused (about 2-3%) around the world, is a major causative agent of chronic liver diseases, including hepatitis, cirrhosis, and hepatocellular carcinoma (HCC) [1, 2]. HCV infection is also associated with lymphoproliferative disorders, represented by mixed type II cryoglobulinemia (MC) and B-cell non-Hodgkin's lymphomas (NHLs) [3–6]. The reason for such different outcome of HCV infection among patients is still largely unknown.

Traditional therapy for treatment of chronic HCV involves pegylated type I IFN (PEG-IFN) in combination with ribavirin. This treatment eradicates HCV infection in only 40%–50% of patients infected with genotypes 1 or 4 and 75%–90% of those infected with genotypes 2 or 3 [7, 8]. However, adverse effects due to this treatment regimen frequently lead to poor tolerance.

Recent studies have reported that human genetic variants, in particular rs1297860 single nucleotide polymorphism (SNP), within or near the interleukin 28B gene (IL28B) are significantly associated with spontaneous HCV clearance as well as different outcomes of treatment with IFN/ribavirin therapy for chronic HCV infection [9–19]. The relation is more evident for HCV genotype 1 infection. These observations suggest an important role of IL28B against HCV infection and natural history of HCV outcomes.

IL28B is a member, along with IL-29 and IL-28A, of type III interferons, also termed interferon-lambda (IFN-*λ*). They have in common a strong antiviral function and induction of interferon stimulated genes (ISGs) [20–24].

IFN-*λ* signals through interferon-lambda receptor1 (IFNLR1) and interleukin-10 receptor2 (IL-10R2) result in STAT phosphorylation, dimer formation, nuclear translocation, and then induction of ISG expression and upregulation of major histocompatibility complex (MHC) class I [25].

IFN-*λ* responsiveness is restricted to specific cell types [26], partially because IFNLR1 tissue distribution is highly restricted [27]. In contrast to the epithelial-like cells, fibroblasts and endothelial cells were completely unresponsive to IFN-*λ*. A good IFN-*λ*-induced response was shown in hepatic cells, with an increased IFNLR1 expression after viral infection [28]. Naïve B and T cells, lymphocytes, and monocytes, although express adequate amounts of IFNLR1 receptor, respond poorly or not at all to IFN-*λ* [28]. This implies the presence of specific mechanisms on the lymphoid tissues that may inhibit the IFN-*λ* response. This important functional difference in tissue response to IFN-*λ* may provide a clinical advantage from IFN-*α* as a treatment for chronic HCV infection, as IFN-*λ* is less likely to induce the leucopenia most often associated with IFN-*α* therapy and may be used in HCV patients who are resistant to IFN-*α*. Moreover, it was found that IFN-*λ* upregulates the production of cytokines by natural killer (NK) and T cells [29] and reduces the proliferation of regulatory T cells [30]; thus [31] it is supposed that lymphoid cells respond to IFN-*λ* by different signals than hepatic cells. Due to the responsiveness of most cell types to IFN-*λ* and a limited toxicity, IFN-*λ*s are under study for clinical use not only for anti-HCV therapy but also as anticancer and antiviral drug [22].

The aim of this paper is to investigate whether a relation between IL28B rs12979860 polymorphism and severity of liver or lymphoproliferative diseases exists.

## 2. Material and Methods

### 2.1. Patients

We identified and reviewed papers published from 2009 to 2013 analyzing Caucasian HCV-infected patients for distribution of IL28B genetic variants, using the online database pubMed. Selected studies included in the analysis along with inclusion criteria were reported in Table 1.

We also analyzed the IL28B rs12979860 polymorphism in a case series of 1050 HCV-infected patients, 47 individuals who experienced spontaneous HCV clearance, and 178 blood donors, from a cohort of HIV- and HBV-negative Italian patients, referred to the following centers: MASVE Center, University of Florence, Florence; “Santa Maria degli Angeli” General Hospital, Pordenone; National Cancer Institute “Fondazione Pascale”, Naples; “Casa Sollievo della Sofferenza” Hospital, San Giovanni Rotondo; “Azienda Ospedaliero-Universitaria Consorziale Policlinico”, Bari; University Hospital “Santa Maria della Misericordia”, Udine; Ospedale san Gerardo-University of Milano-Bicocca, Monza; and National Cancer Institute “Centro di Riferimento Oncologico”, CRO Aviano National Cancer Institute. Our series of 1050 patients with chronic HCV infection included group of patients with absence of HCC or any sign/symptom of definite MC or NHL (CHC, *n* = 536), patients with HCC (*n* = 95), patients with a definite MC syndrome, according to previously described criteria [33] (*n* = 352), and presence of a definite malignant B-cell NHL (*n* = 67).

### 2.2. IL28B Genotyping

Genomic DNA was extracted from peripheral blood which has been previously stored at −80°C.

IL28B genotyping was performed using a specific customized TaqMan SNP-genotyping Assay (rs12979860; Applied Bosystem, Foster City, CA, USA) based on allele-specific labeled probes on a Rotor Gene 6000 (Corbett Research, Sidney, Australia). Amplicon sequencing was used to validate the genotyping techniques.

### 2.3. Statistical Analysis

Frequencies (f) of IL28B rs12979860 C and T alleles and genotypes polymorphisms were determined individually by direct counting of the positive individuals for a specific genotype/allele polymorphism. The observed and expected genotype frequencies among the study groups were analyzed using the Hardy-Weinberg equilibrium theory. Differences in f between groups of selected patients were evaluated using Fisher's exact test, Pearson's chi-square (*χ* ^2^) test (with Yates correction), or Cochran-Armitage *z* ^2^ test for trend in frequencies as appropriate [34]. A two-sided *P* value <0.05 was considered as statistically significant.

## 3. Results

**Figure 1:**
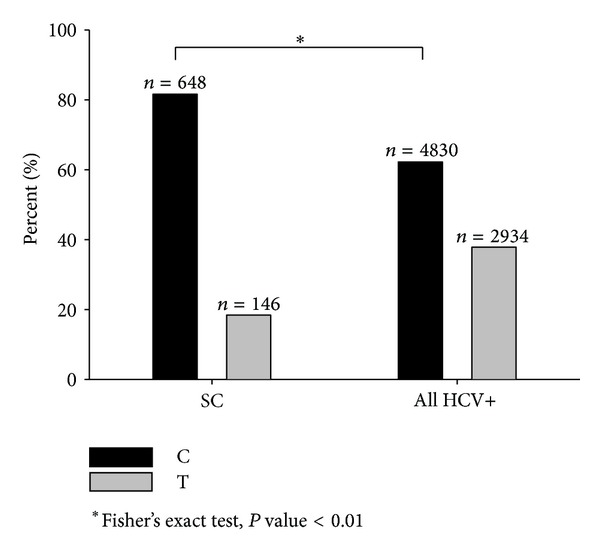
IL28B rs12979860 C/T allele frequencies in patients with spontaneous clearance (SC) of HCV infection versus all HCV+ patients. Caucasian individuals reported in literature (PubMed), 2009–2013.

Reviewed data on IL28B variant distribution in different HCV outcomes and limited to Caucasian subjects were reported in Table 2. The genotype frequencies of the IL28B rs12979860 polymorphism were found in Hardy-Weinberg equilibrium in the blood donors group (Pearson's *χ* ^2^ = 0.09; df = 1; *P* = 0.76). The IL28B rs12979860 C allele was related to a significant increase in spontaneous clearance of the virus in a dominant genetic model (CC versus CT + TT) employed to compare HCV+ patients presenting spontaneous viral clearance versus all other HCV+ subjects (Pearson's *χ* ^2^ = 148.9; *P* < 0.001). Data indicates that C allele had an important role in the control of infection (Fisher's test, *P* < 0.001, Figure 1).

**Figure 2:**
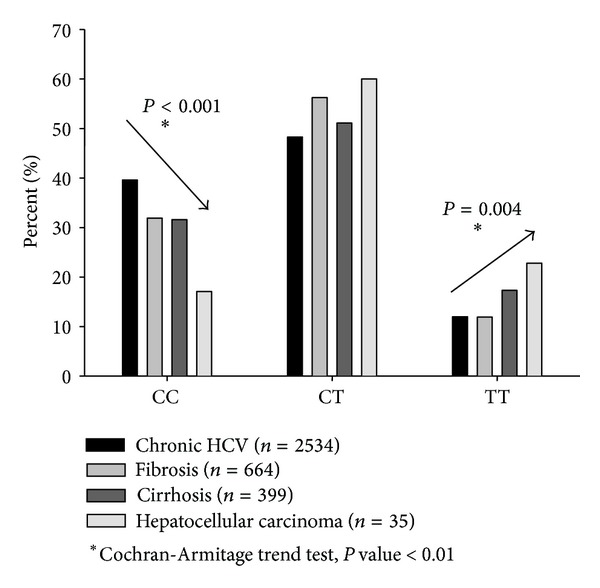
IL28B rs12979860 genotype frequencies in patients with HCV infection and different outcomes. Caucasian individuals reported in literature (PubMed), 2009–2013.

A significant negative linear trend was detected in the order of the CC genotype frequencies of patients with an increasing severity of liver diseases (from 40% in CHC to 17% in HCC, Figure 2; Cochran-Armitage's *z* ^2^ = 22.38, *P* < 0.001), whereas a significant positive linear trend emerged for TT genotype frequencies (from 12.0% in CHC to 22.8% in HCC, Figure 2; Cochran-Armitage's *z* ^2^ = 8.42, *P* = 0.004), suggesting an important role of CC allele in the control of liver disease progression.

**Figure 3:**
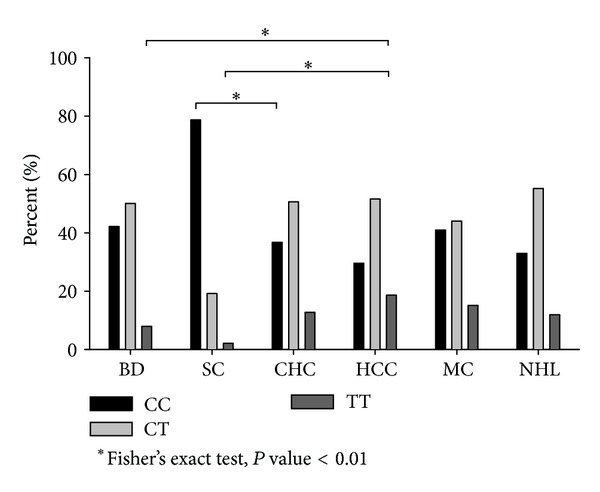
Genotype frequencies of IL28B rs12979860 C/T polymorphism in 178 blood donors (BD), 47 patients with spontaneous clearance (SC) of HCV, 536 patients with chronic HCV (CHC), 95 patients with hepatocellular carcinoma (HCC), 352 patients with mixed cryoglobulinemia syndrome (MC), and 67 patients with non-Hodgkin's lymphoma (NHL). Italian case series.

IL28B rs12979860 genotype frequencies found in our case series were similar to those reported in the literature (Table 3). We found a slightly increasing trend in TT genotype frequency in HCC (18.9%) with respect to that of CHC (12.7%), but difference was not statistically significant (Fisher's test, *P* = 0.106) (Figure 3). Given the modest increase in HCC risk we found and the low number of HCC cases included in this study (35 from the literature and 95 in our case series), replication studied using larger series of HCV+ HCC would need to confirm this finding. However, carriage of TT genotype in blood donors (7.9%) and in patients who spontaneously cleared the virus (2.1%) was lower than that found in our HCC series (Fisher's test, *P* = 0.009 and 0.003, resp.). Moreover, similar to that found in data from the literature, the frequency of CC genotype was significantly higher among patients who spontaneously cleared the virus compared to patients with CHC (Fisher's test, *P* < 0.001) (Table 3 and Figure 3).

As regards the lymphoproliferative disorders associated with HCV infection, the literature reported only one study including patients with autoimmune MC syndrome [15] and no study on NHL. Data obtained in our case series were reported in Table 3.

In MC, IL28B CC genotype frequency was found similar to that in blood donors (Figure 3); nonetheless, the carriage of TT genotype in MC (15.1%) was weakly higher than that among blood donors (7.9%, Fisher's test, *P* = 0.019) but not significantly higher than that among CHC patients (12.9, Fisher's test, *P* = 0.319). HCV positive patients with NHL (11.9%) were very similar to CHC patients; in this case the carriage of TT genotype was not significantly different.

## 4. Discussion

Several studies have indicated that IL28B rs12979860 polymorphism has a role in the response of HCV infection and liver disease risk, but the knowledge of its overall effect on the natural history of hepatitis viral infection remains partial and limited to certain aspect in individual studies. In addition, data regarding the IL-28B rs12979860 risk towards a HCV-related lymphoproliferative disorder (MC or NHL) is even less known. Thus we conducted this study to evaluate relations between IL-28B rs12979860 CT polymorphism and natural history of HCV infection by using the following inclusion criteria: Caucasian individuals affected by HCV infection (excluding HBV or HIV co-infection) and different liver or lymphoproliferative diseases related to HCV progression. The analysis of aggregated data from the literature clearly indicated that IL-28B rs12979860 allelic frequencies were significantly different in patients who spontaneously cleared HCV infection in comparison to patients with chronic HCV infection. HCV+ patients who spontaneously cleared the infection carried the CC genotype in 68.8% of cases, while patients with chronic HCV infection and liver diseases carried this genotype in a range from 39.6% to 17.1%. The IL-28B C allele was inversely related to the risk of chronic and liver diseases with respect to both blood donors and patients who spontaneously eliminated the virus when the dominant genetic model (CC versus CT + TT) was employed, in agreement with previous idea that C allele is implicated in the control of HCV infection. In addition, although resulting from a limited number of cases analyzed (*n* = 35 from literature [32], *n* = 95 in our series), the carriage of TT genotype and the presence of T allele were slightly prevalent in the HCC group of patients. Due to the weak effect of IL28B T allele found on the progression to HCC, replication of this analysis in a large number of cases is required to confirm our study finding. Anyhow, this finding is in accordance with the HCC carcinogenic model where IL28B T allele carriage was much more favorable to persistence of a chronic HCV infection, which leads to both hepatocyte injury and chronic inflammation in the liver, thus facilitating the HCC development [35–37].

We presented for the first time a comparative analysis of IL28B rs12979860 allelic distribution in patients with chronic HCV infection and lymphoproliferative diseases (Table 3 and Figure 3). No statistically significant differences in IL28B rs12979860 allelic distribution emerged between patients with lymphoproliferative disorders and other groups of patients or blood donors. It should be noted, however, that CC genotype was more frequent in patients with MC (144/352, 40.9%) than in those with malignant tumors taken together (HCC + NHL, 50/162, 30.9%, Fisher's test *P* = 0.031, data not shown in Tables). Moreover, TT genotype was slightly more frequent, without reaching statistical significance maybe because of low numbers, in patients with an HCC (18.9%) than in those with a NHL (11.9%, Figure 3). These findings could suggest that patients with MC syndrome carrying IL28B rs12979860 CC, a genotype more favorable to resolve the infection, should contrast both liver and lymphoproliferative malignant diseases and that patients with TT genotype could be more predisposed to evolve towards an HCC than a malignant lymphoma.

In conclusion, study results on overall data from the literature and our case series confirmed that all chronic HCV-related disorders presented a lower frequency of C allele as compared to patients who spontaneously eliminated HCV, thus supporting the model of persistent HCV infection as an important risk factor for the pathogenesis of both liver and lymphoproliferative disorders [3, 6]. Moreover, an additional risk factor related to TT homozygosis and liver disease progression emerged from this study. Of note, since recently a polymorphism (ss469415590) of a new interferon gene, IFNL4, was found strongly associated with HCV clearance and was in high linkage disequilibrium with IL28B [38]; further studies are needed regarding IFNL4 polymorphism and its possible involvement in the HCV-related malignancies.

## Figures and Tables

**Table 1:** Caucasian patients included in IL28B rs12979860 analysis with different HCV-related outcomes reported in the literature (PubMed) between 2009 and 2013.

Article	Blood donors (HCV neg.)	Spontaneous clearance of HCV	Chronic HCV	Mild/moderate fibrosis	Cirrhosis	Hepatocellular carcinoma	Mixed cryoglobulinemia S.
Mangia et al. (2013) [13]	178	47	122				
Sharafi et al. (2012) [17]	142		921				
Piluso et al. (2013) [15]			231				250
Falleti et al. (2011) [11]	428			429	200		
Knapp et al. (2011) [12]	74	89	234				
Fabris et al. (2011) [32]	344			235	199	35	
Sarrazin et al. (2011) [16]	200		645				
Thomas et al. (2009) [18]		261	381				

Total	1366	397	2534	664	399	35	250

**Table 2:** IL28B rs12979860 variant frequencies in blood donors and patients with HCV infection and different outcomes. Caucasian individuals reported in literature (PubMed), 2009–2013.

	Blood donors (HCV neg.)	Spontaneous clearance of HCV	HCV patients					HCV patients total
			Chronic HCV	Mild/moderate fibrosis	Cirrhosis	Hepatocellular carcinoma	Mixed cryoglobulinemia S.	
	Number (%)	Number (%)	Number (%)	Number (%)	Number (%)	Number (%)	Number (%)	Number (%)
IL28B genotype								
CC	631 (46.2)	273 (68.8)	1004 (39.6)	212 (31.9)	126 (31.6)	6 (17.1)	99 (39.6)	1447 (37.3)
CT	599 (43.8)	102 (25.7)	1225 (48.3)	373 (56.2)	204 (51.1)	21 (60.0)	113 (45.2)	1936 (49.9)
TT	136 (10.0)	22 (5.5)	305 (12.0)	79 (11.9)	69 (17.3)	8 (22.8)	38 (15.2)	499 (12.8)
Total	**1366 (100.0)**	**397 (100.0)**	**2534 (100.0)**	**664 (100.0)**	**399 (100.0)**	**35 (100.0)**	**250 (100.0)**	**3882 (100.0)**
*χ* ^2^ (*P* value)^a^	—	Ref.	118.8 (<0.001)	135.9 (<0.001)	112.4 (<0.001)	40.5 (<0.001)	55.7 (<0.001)	148.9 (<0.001)

IL28B dominant model								
CC	631 (46.2)	273 (68.8)	1004 (39.6)	212 (31.9)	126 (31.6)	6 (17.1)	99 (39.6)	1447 (37.3)
CT + TT	735 (53.8)	124 (31.2)	1530 (60.4)	452 (68.1)	273 (68.4)	29 (82.9)	151 (60.1)	2435 (62.7)
Total	**1366 (100.0)**	**397 (100.0)**	**2534 (100.0)**	**664 (100.0)**	**399 (100.0)**	**35 (100.0)**	**250 (100.0)**	**3882 (100.0)**
*χ* ^2^ (*P* value)^a^	—	Ref.	118.6 (<0.001)	135.9 (<0.001)	110.1 (<0.001)	37.5 (<0.001)	53.4 (<0.001)	148.6 (<0.001)

IL28B allele								
C	1861 (68.1)	648 (81.6)	3233 (63.8)	797 (60.0)	456 (57.1)	33 (47.1)	311 (62.2)	4830 (62.2)
T	871 (31.9)	146 (18.4)	1835 (36.2)	531 (40.0)	342 (42.9)	37 (52.9)	189 (37.8)	2934 (37.8)
Total	**2732 (100.0)**	**794 (100.0)**	**5068 (100.0)**	**1328 (100.0)**	**798 (100.0)**	**70 (100.0)**	**500 (100.0)**	**7764 (100.0)**
*χ* ^2^ (*P* value)^a^	—	Ref.	97.4 (<0.001)	106.7 (<0.001)	112.1 (<0.001)	45.8 (<0.001)	60.3 (<0.001)	117.7 (<0.001)

^a^Pearson's chi-square with spontaneous clearance of HCV as reference group.

**Table 3:** IL28B rs12979860 variant frequencies in 178 blood donors and 1097 patients with HCV infection and different outcomes. Italian case series.

	Blood donors (HCV neg.)	Spontaneous clearance of HCV	HCV patients				HCV patients total
			Chronic HCV	Hepatocellular carcinoma	Mixed cryoglobulinemia S.	Non-Hodgkin's lymphoma	
	Number (%)	Number (%)	Number (%)	Number (%)	Number (%)	Number (%)	Number (%)
IL28B genotype							
CC	75 (42.1)	37 (78.7)	197 (36.7)	28 (29.5)	144 (40.9)	22 (32.9)	391 (37.2)
CT	89 (50.0)	9 (19.2)	271 (50.6)	49 (51.6)	155 (44.0)	37 (55.2)	512 (48.8)
TT	14 (7.9)	1 (2.1)	68 (12.7)	18 (18.9)	53 (15.1)	8 (11.9)	147 (14.0)
Total	**178 (100.0)**	**47 (100.0)**	**536 (100.0)**	**95 (100.0)**	**352 (100.0)**	**67 (100.0)**	**1050 (100.0)**
*χ* ^2^ (*P* value)^a^	—	Ref.	31.9 (<0.001)	31.4 (<0.001)	24.4 (<0.001)	23.5 (<0.001)	32.8 (<0.001)

IL28B dominant model							
CC	75 (42.1)	37 (78.7)	197 (36.7)	28 (29.5)	144 (40.9)	22 (32.9)	391 (37.2)
CT + TT	103 (57.9)	10 (21.3)	339 (63.3)	67 (70.5)	208 (59.1)	45 (67.1)	659 (62.8)
Total	**178 (100.0)**	**47 (100.0)**	**536 (100.0)**	**95 (100.0)**	**352 (100.0)**	**67 (100.0)**	**1050 (100.0)**
*χ* ^2^ (*P* value)^a^	—	Ref.	31.7 (<0.001)	30.7 (<0.001)	23.9 (<0.001)	23.3 (<0.001)	32.5 (<0.001)

IL28B allele							
C	239 (67.1)	83 (88.3)	665 (62.0)	105 (55.3)	443 (62.9)	81 (60.4)	1294 (61.2)
T	117 (32.9)	11 (11.7)	407 (48.0)	85 (44.7)	261 (37.1)	53 (39.6)	806 (38.8)
Total	**356 (100.0)**	**94 (100.0)**	**1072 (100.0)**	**190 (100.0)**	**704 (100.0)**	**134 (100.0)**	**2100 (100.0)**
*χ* ^2^ (*P* value)^a^	—	Ref.	25.9 (<0.001)	30.7 (<0.001)	23.8 (<0.001)	21.2 (<0.001)	27.4 (<0.001)

^a^Pearson's chi-square with spontaneous clearance of HCV as reference group.
